# Immune checkpoint blockade and thrombo-inflammatory remodeling in microsatellite instability-high intrahepatic cholangiocarcinoma with Trousseau syndrome: a case report

**DOI:** 10.3389/fmed.2026.1937471

**Published:** 2026-09-18

**Authors:** XiaoXiao Sun, XiaoTong Kuang, ShanShan Sun, Yan Cui, Xiao Xu, JiGang Dong, Fang Lin

**Affiliations:** 1 Department of Radiation Oncology, Jiaozhou Central Hospital of Qingdao, Qingdao, China; 2 Department of Science Education, Jiaozhou Central Hospital of Qingdao, Qingdao, China; 3 Department of Health Management, Jiaozhou Central Hospital of Qingdao, Qingdao, China

**Keywords:** cancer-associated thrombosis, immune checkpoint inhibitor, intrahepatic cholangiocarcinoma, thrombo-inflammation, Trousseau syndrome

## Abstract

**Background:**

Cancer-associated thrombosis (CAT) is a major cause of morbidity and mortality in patients with malignancy and is increasingly recognized as a manifestation of tumor-associated thrombo-inflammation. Although immune checkpoint inhibitors (ICIs) have transformed the treatment of microsatellite instability-high (MSI-H) malignancies, their impact on the interplay between antitumor immunity and cancer-associated thrombosis remains poorly understood, particularly in intrahepatic cholangiocarcinoma (ICC).

**Case presentation:**

We report a 74-year-old woman with MSI-H intrahepatic cholangiocarcinoma presenting with Trousseau syndrome characterized by extensive arterial and venous thrombotic events, including deep vein thrombosis, portal vein thrombosis, multifocal cerebral infarctions, splenic infarction, and renal infarction. Following treatment with the PD-L1 inhibitor envafolimab in combination with rivaroxaban, radiographic evaluation demonstrated stable disease without recurrent thrombotic events. Longitudinal biomarker assessment revealed temporally associated reductions in circulating D-dimer and interleukin-6 (IL-6) concentrations during treatment, while peripheral immune-cell subsets exhibited dynamic changes that paralleled systemic inflammatory activity. Despite a temporary interruption of immunotherapy because of influenza A-associated pneumonia, both inflammatory and coagulation markers declined following resolution of the infection.

**Conclusion:**

This case underscores the complex interplay between systemic inflammation, coagulation activation, and tumor progression in MSI-H intrahepatic cholangiocarcinoma with Trousseau syndrome. Serial assessment of coagulation and inflammatory biomarkers may help characterize disease dynamics and inform individualized management.

## 1 Introduction

Intrahepatic cholangiocarcinoma (ICC) is the second most common primary liver malignancy and remains one of the most aggressive hepatobiliary cancers, with limited therapeutic options and poor long-term survival in patients with advanced disease (1). Although microsatellite instability-high (MSI-H) or mismatch repair-deficient (dMMR) tumors account for only approximately 1.7%–3.1% of ICC cases, this molecular subtype has emerged as a clinically important subgroup because of its marked sensitivity to immune checkpoint inhibitors (ICIs). Consequently, immunotherapy has become an important treatment option for patients with advanced MSI-H ICC, offering durable responses in a subset of patients who had few effective systemic therapies.

Beyond tumor progression, cancer-associated thrombosis (CAT) represents a major determinant of morbidity and mortality in patients with solid malignancies and constitutes the second leading cause of non-cancer-related death (2, 3). Increasing evidence indicates that CAT should no longer be regarded as a simple coagulation disorder but rather as a thrombo-inflammatory syndrome arising from dynamic interactions among tumor cells, immune cells, vascular endothelium, platelets, and the coagulation system. Within this network, inflammatory cytokines—particularly interleukin-6 (IL-6)—occupy a central regulatory position by promoting endothelial activation, inducing tissue factor expression, enhancing platelet activation, and facilitating neutrophil extracellular trap (NET) formation, thereby establishing and sustaining a systemic hypercoagulable state (4). This integrated thrombo-inflammatory model provides a biological framework linking tumor progression with thrombotic complications (5).

ICIs have fundamentally reshaped cancer treatment by restoring antitumor immunity through blockade of inhibitory immune checkpoints. However, their influence on CAT remains incompletely understood. On one hand, immune activation induced by ICIs has been proposed to increase thrombotic risk through endothelial injury, cytokine release, and immune-mediated vascular inflammation. Conversely, accumulating clinical observations suggest that effective immune checkpoint blockade may attenuate tumor-driven inflammation, thereby indirectly mitigating the hypercoagulable state in selected patients (6). These apparently conflicting findings imply that the net effect of ICIs on thrombosis is likely determined by the balance between immune activation and suppression of tumor-associated thrombo-inflammatory signaling (7, 8).

This question may be particularly relevant in MSI-H malignancies, where highly immunogenic tumors exhibit profound immune remodeling following PD-1/PD-L1 blockade. Nevertheless, clinical evidence describing the concurrent evolution of tumor response, systemic inflammation, and thrombotic activity in MSI-H ICC remains extremely limited. Whether successful immunotherapy can simultaneously modulate tumor progression and cancer-associated thrombosis through shared immunological mechanisms has not been adequately explored.

We describe a patient with MSI-H intrahepatic cholangiocarcinoma presenting with Trousseau syndrome characterized by extensive arterial and venous thromboses. Following treatment with envafolimab combined with anticoagulation, radiographic disease stabilization was accompanied by parallel reductions in serum IL-6 and D-dimer concentrations without recurrent thrombotic events. This case provides a temporal association between tumor immune remodeling and thrombo-inflammatory activity and raises the possibility that attenuation of inflammation-driven hypercoagulability may accompany effective immune checkpoint blockade.

## 2 Case presentation

A 74-year-old woman was hospitalized in October 2025 after experiencing intermittent back pain. Her medical history included coronary artery disease and prior cholecystectomy. Laboratory testing on admission revealed high-sensitivity troponin I 0.1000 ng/mL (reference range: < 0.0313 ng/mL) and an elevated D-dimer level of 1.76 mg/L (reference range: < 0.50 mg/L). Lower-extremity venous ultrasonography demonstrated thrombosis of the bilateral calf intermuscular veins, with non-compressible venous lumens and absent significant blood-flow signals on color Doppler imaging.

Subsequent contrast-enhanced CT identified a hepatic mass located in the right lobe with evidence of portal vein involvement and enlarged abdominal lymph nodes. Further abdominal pelvic computed tomography (APCT) revealed multiple systemic thrombotic events, including splenic infarction, and infarction of the left kidney (Figure 1). Marked hypercoagulability and multiple systemic thrombotic and ischemic events prompted intracranial evaluation after discussion with the patient and her family. Brain MRI demonstrated multifocal cerebral infarctions involving different vascular territories (Figure 2). Immunohistochemistry showed CK7 (+), CK20 (−), Villin (+), CDX2 (−), Ki-67 approximately 20%, wild-type p53 expression, HER2 (0), AFP (−), Hepat-1 (−), and CK19 (+). The tumor demonstrated an atypical MMR immunophenotype, with retained MLH1 and MSH6 expression and loss of MSH2 and PMS2 expression. PCR-based microsatellite instability testing confirmed an MSI-high phenotype. These findings supported the diagnosis of dMMR/MSI-H intrahepatic cholangiocarcinoma. Given the patient's advanced age and absence of a known family history suggestive of Lynch syndrome, germline testing was not performed; therefore, a hereditary MMR defect cannot be definitively excluded. After multidisciplinary team (MDT) discussion involving physicians from Radiology, Hepatobiliary Surgery, Neurology, and Pathology, the diagnosis is confirmed as intrahepatic cholangiocarcinoma (adenocarcinoma, dMMR/MSI-H, cT2N1M1, Stage IV) in association with Trousseau syndrome. The patient-initiated treatment with subcutaneous envafolimab (150 mg weekly) on October 30, 2025, in combination with rivaroxaban (20 mg daily).

**Figure 1:**
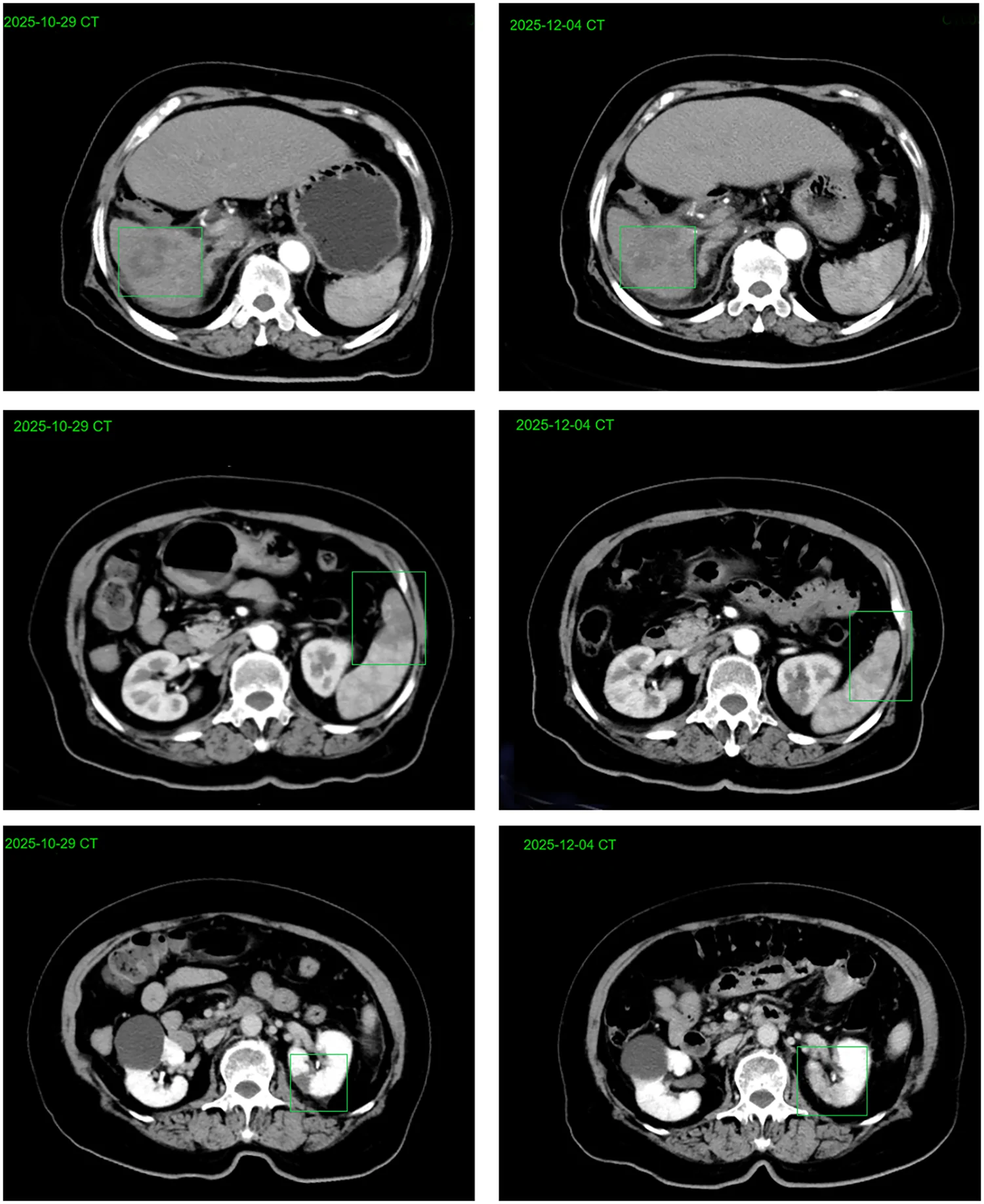
Radiologic findings of intrahepatic cholangiocarcinoma and systemic thrombotic complications during treatment**.** Contrast-enhanced abdominal computed tomography (APCT) performed at diagnosis demonstrated a hepatic mass in the right liver lobe with portal vein invasion and abdominal lymph node metastases, accompanied by multiple thrombotic complications, including splenic infarction and left renal infarction. Follow-up APCT after treatment with envafolimab plus rivaroxaban showed stable tumor burden without evidence of newly developed thrombotic lesions, consistent with radiographic stable disease.

**Figure 2:**
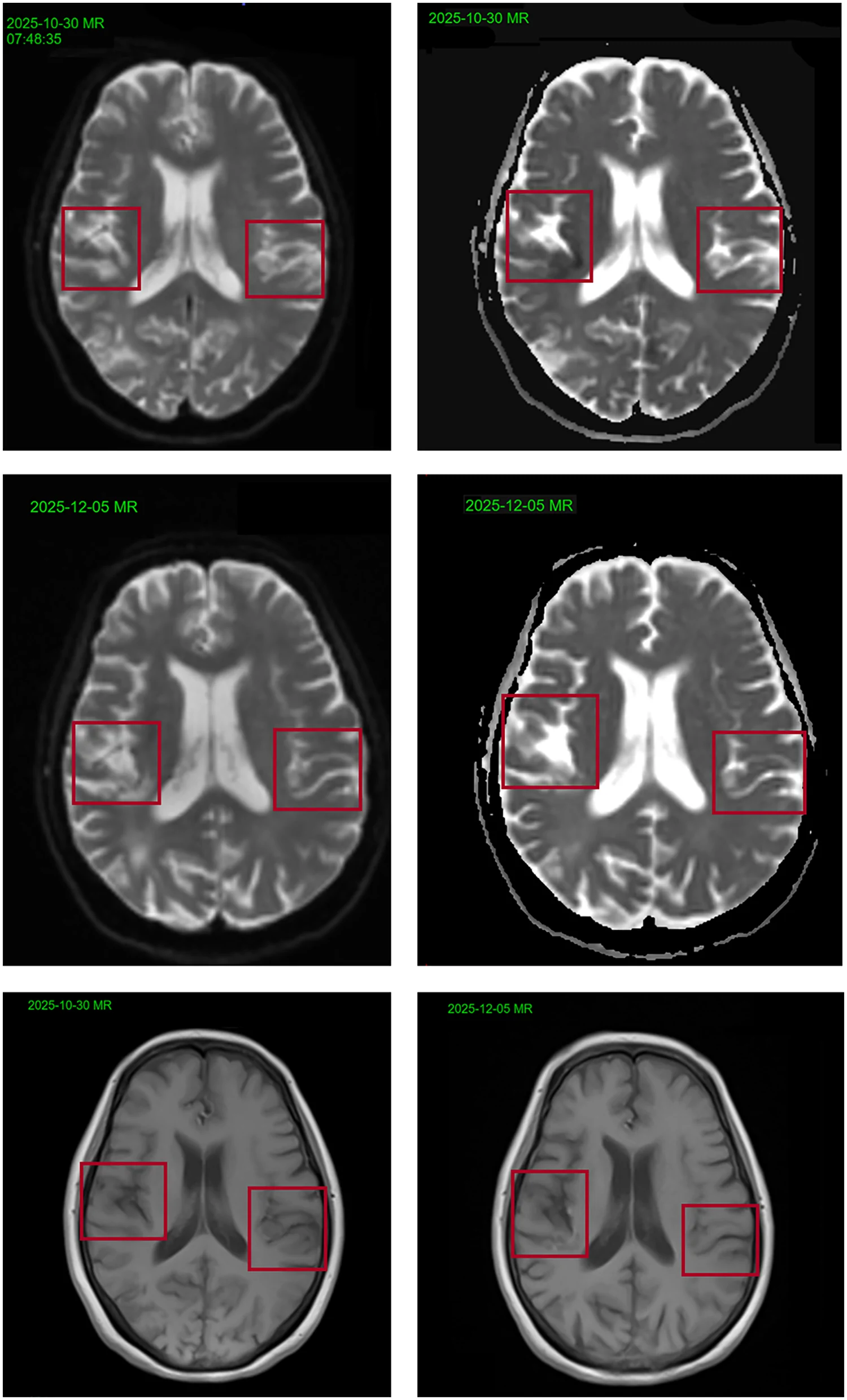
Dynamic changes in cerebral ischemic lesions during the disease course. Diffusion-weighted imaging (DWI) performed at presentation demonstrated multiple acute cerebral infarctions involving different vascular territories, consistent with systemic thromboembolic disease. Follow-up MRI obtained during treatment showed no newly developed ischemic lesions.

In late December 2025, the patient developed influenza A-associated pneumonia with high fever (39.1 °C), acute kidney injury (creatinine 65→211 μmol/L), and marked inflammatory activation. The patient had to temporarily discontinue immunotherapy and instead receive corticosteroids and supportive care. Given the acute renal impairment, rivaroxaban was temporarily switched to unfractionated heparin after multidisciplinary consultation. Renal function rapidly recovered, with serum creatinine decreasing to 101 μmol/L within 3 days and subsequently continuing to decline, after which rivaroxaban 20 mg once daily was resumed. Following treatment, the pneumonia improved and systemic inflammation markedly subsided.

During treatment, CA19-9 decreased from >10,000 U/mL to 8,346 U/mL but remained markedly elevated. APCT in December 2025 demonstrated stable disease, while cranial MRI showed no significant progression of the cerebral lesions (Figures 1, 2). Importantly, no new thrombotic events occurred during this period. Serial measurements revealed dynamic changes in coagulation, inflammatory, and immune-related parameters throughout the disease course (Figure 3). D-dimer and interleukin-6 (IL-6; reference range: 0.00-7.01 pg/mL) levels exhibited broadly parallel temporal patterns (Figure 3A). Following initiation of immune checkpoint inhibition, D-dimer levels gradually declined from a peak value of 3.89 mg/L (2025-12-08) to 0.43 mg/L (2026-01-14) (∼89% reduction). IL-6 fluctuated during lung infection, peaking at 140.71 pg/mL (2025-12-29) and subsequently declining to 3.74 pg/mL (2026-01-14). Systemic inflammatory indices demonstrated relatively modest fluctuations over time. The neutrophil-to-lymphocyte ratio (NLR) and platelet-to-lymphocyte ratio (PLR) remained low throughout the observation period, whereas the lymphocyte-to-monocyte ratio (LMR) showed a gradual increase before transiently peaking and subsequently returning toward baseline (Figure 3B). Analysis of lymphocyte subsets demonstrated dynamic alterations accompanying changes in inflammatory activity (Figure 3C). Periods of elevated IL-6 levels were associated with a reduction in the proportion of peripheral CD4⁺ T cells, declining from approximately 34% to 26%, whereas the proportion of natural killer (NK) cells increased from approximately 16% to 37%. An inverse temporal trend between serum IL-6 concentrations and peripheral CD4⁺ T-cell proportions was observed during follow-up (Figure 3D).

**Figure 3:**
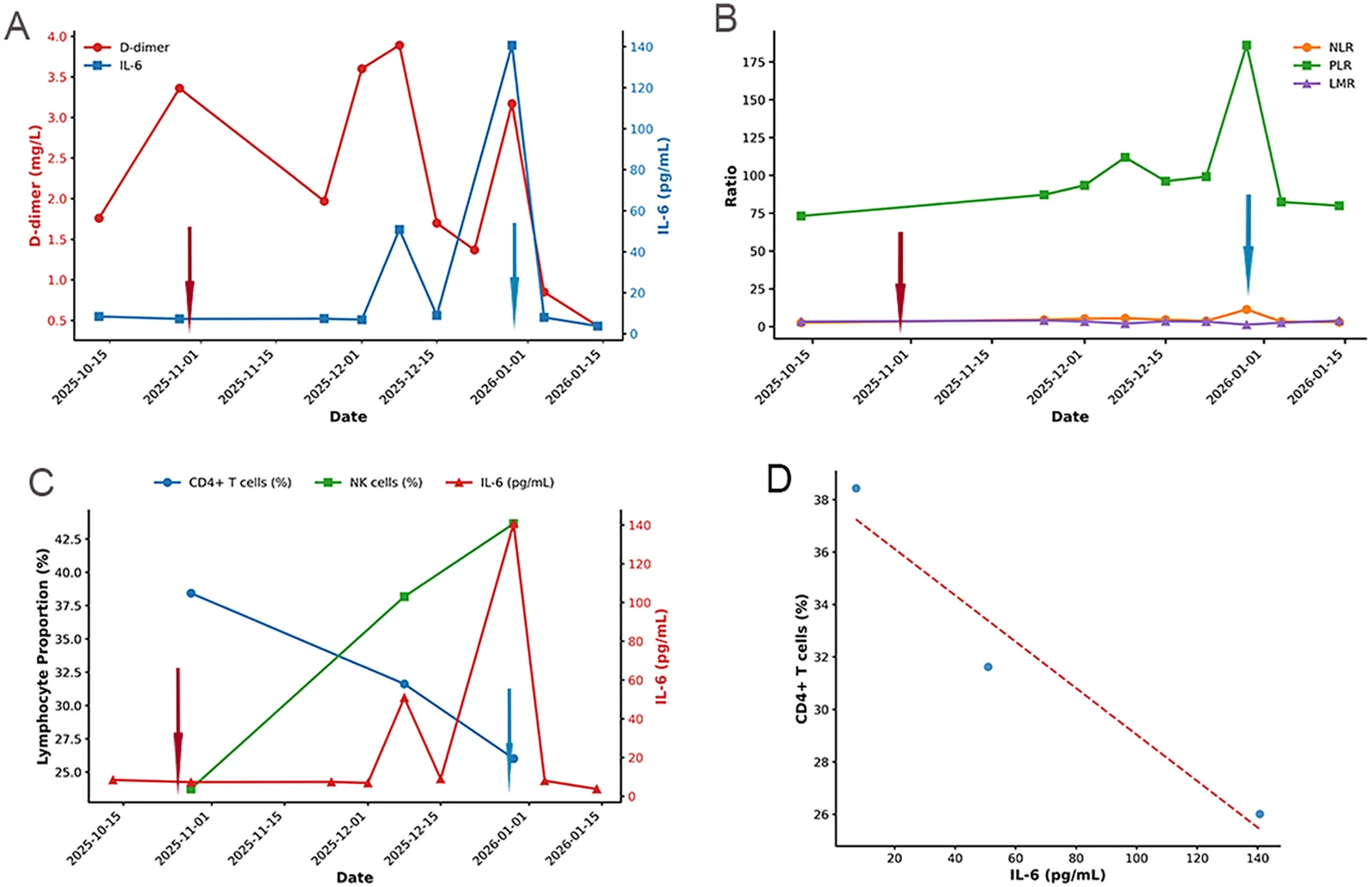
Longitudinal thrombO,inflammatory and immune profiling during treatment with immune checkpoint inhibition. Red arrows indicate the initiation of anticoagulation and antitumor therapy on October 30, 2025. The blue arrow indicates the onset of influenza A-associated pneumonia. **(A)** Dynamic changes in D-dimer and interleukin-6 (IL-6) levels over time. The left Y-axis represents D-dimer concentrations (mg/L, red line), while the right *Y*-axis indicates serum IL-6 levels (pg/mL, blue line). IL-6, interleukin-6. **(B)** Temporal changes in systemic inflammatory indices. The line graph illustrates longitudinal variations in the neutrophil-to-lymphocyte ratio (NLR, orange), platelet-to-lymphocyte ratio (PLR, green), and lymphocyte-to-monocyte ratio (LMR, purple) during the disease course. NLR, neutrophil-to-lymphocyte ratio; PLR, platelet-to-lymphocyte ratio; LMR, lymphocyte-to-monocyte ratio. **(C)** Temporal association between IL-6 levels and lymphocyte subsets. The left *Y*-axis indicates the peripheral proportions of CD4+ T cells (blue line) and natural killer (NK) cells (green line). In contrast, the right *Y*-axis represents serum IL-6 concentrations (pg/mL, red line). IL-6, interleukin-6; NK, natural killer. **(D)** Temporal changes in serum IL-6 and peripheral CD4⁺ T-cell proportions. An inverse temporal pattern was observed between the two parameters. Given the limited number of longitudinal observations, this finding is presented as an exploratory trend. IL-6, interleukin-6.

However, due to personal reasons, the patient did not return to the hospital for follow-up visits or continue immunotherapy as scheduled, and only received oral rivaroxaban anticoagulant therapy. On March 20, 2026, the patient suddenly died at home, the emergency physician diagnosed a cerebrovascular or thromboembolic event.

## 3 Discussion

The present case illustrates the temporal relationship between tumor control, systemic inflammation, and cancer-associated thrombosis in a patient with MSI-H intrahepatic cholangiocarcinoma. Rather than evolving as independent clinical events, tumor burden, inflammatory activity, and coagulation status exhibited temporally associated changes throughout treatment. Following PD-L1 blockade with envafolimab, radiographic disease stabilization was accompanied by progressive reductions in circulating IL-6 and D-dimer concentrations without recurrent thrombotic events. Although causality cannot be established from a single observation, the longitudinal association observed here provides a clinically informative, hypothesis-generating observation regarding the potential interplay between tumor immunity and thrombo-inflammatory activity.

Cancer-associated thrombosis is increasingly recognized as a manifestation of tumor-driven thrombo-inflammation rather than a disorder solely resulting from activation of the coagulation cascade. Malignant cells actively reshape the systemic immune microenvironment by releasing inflammatory cytokines, extracellular vesicles, and procoagulant mediators, thereby establishing reciprocal interactions among immune cells, endothelial cells, platelets, and coagulation pathways. Among these mediators, IL-6 occupies a central position, functioning as a molecular bridge between chronic inflammation and thrombosis (9). Experimental studies have demonstrated that IL-6 promotes endothelial activation, induces tissue factor expression on monocytes and endothelial cells, enhances platelet activation, and facilitates neutrophil extracellular trap (NET) formation, ultimately amplifying thrombin generation and fibrin deposition (10). These interconnected processes constitute the biological basis of Trousseau syndrome and explain why thrombotic events frequently parallel tumor progression.

The influence of immune checkpoint inhibitors on thrombosis, however, remains controversial. Previous observational studies have reported inconsistent results, with some suggesting an increased incidence of arterial or venous thromboembolic events during immune checkpoint blockade, whereas others have demonstrated no significant increase or even potential improvement in thrombotic risk (8, 11, 12). These discrepancies are unlikely to reflect contradictory biological effects of ICIs themselves. Instead, they probably arise from differences in tumor biology, baseline inflammatory status, treatment response, and patient selection (12–14). Immune checkpoint blockade simultaneously activates antitumor immunity while also remodeling the tumor microenvironment; therefore, its overall impact on coagulation is expected to depend on the dynamic balance between immune activation and suppression of tumor-derived inflammatory signaling (11, 15–17).

This concept may be particularly relevant in MSI-H malignancies, which are characterized by a high tumor mutational burden, abundant neoantigen expression, and pre-existing immune infiltration. Restoration of exhausted T-cell function following PD-1/PD-L1 blockade often produces profound remodeling of the immune microenvironment, resulting in suppression of tumor-promoting inflammatory circuits in responders (15, 16). In the present patient, disease stabilization was accompanied by a sustained decline in IL-6 levels after resolution of influenza-associated pneumonia. Importantly, D-dimer decreased in parallel while anticoagulation with rivaroxaban was continued, suggesting that attenuation of the hypercoagulable state may have been temporally associated with reduced tumor-associated inflammatory activity rather than being attributable to anticoagulation alone. This temporal relationship is biologically plausible because effective tumor control may diminish continuous cytokine production, thereby interrupting the feed-forward cycle linking inflammation, endothelial activation, coagulation, and thrombosis. However, D-dimer is a nonspecific marker of coagulation activation, and the concurrent clinical changes during follow-up preclude attribution of its decline to ICI therapy alone.

An additional finding deserving attention was the dynamic alteration of peripheral immune-cell subsets. Periods of elevated IL-6 concentrations coincided with a relative reduction in circulating CD4⁺ T-cell proportions and an expansion of NK cells, whereas normalization of IL-6 was accompanied by stabilization of immune-cell composition. Although the limited number of sampling time points precludes definitive conclusions, these observations are consistent with a link between systemic inflammation and immune remodeling. Longitudinal immune profiling with functional T-cell phenotyping, cytokine profiling, and single-cell analysis is warranted to validate these observations.

Collectively, the clinical course observed in this patient supports a cautious working model in which tumor-associated inflammation may contribute to cancer-associated thrombosis, while successful immune checkpoint blockade may be temporally associated with attenuation of thrombo-inflammatory signaling through effective tumor control and immune remodeling. IL-6-centered inflammatory networks may promote endothelial activation, tissue factor expression, platelet activation, and NET formation, thereby contributing to systemic hypercoagulability. Cancer-associated thrombosis may therefore represent an immunologically regulated component of tumor biology that evolves alongside antitumor treatment response. However, this hypothesis remains unproven, and the present case does not establish a direct antithrombotic effect of ICI therapy.

Emerging experimental evidence provides additional mechanistic context for the potential interaction between coagulation and antitumor immunity. Kött et al. recently summarized preclinical and clinical evidence suggesting that coagulation factors, factor Xa signaling, and platelet pathways may influence the tumor microenvironment and immune responses to immune checkpoint blockade. Importantly, their review indicated that evidence for such interactions is stronger in preclinical models than in clinical studies, underscoring the need for prospective validation before these observations can be translated into therapeutic strategies (18). Clinical observations provide similarly suggestive but non-definitive evidence. In a multicenter ADOReg study of 2,419 patients with advanced melanoma receiving ICI therapy, concomitant antithrombotic treatment was associated with longer progression-free survival, while certain anticoagulant regimens, including DOACs, were associated with lower mortality in multivariable analyses (19). These findings provide clinical context for a potential interaction between coagulation-related pathways and ICI efficacy.

The patient's subsequent clinical course provides an important counterpoint to the relative stability observed during ICI treatment. ICI was discontinued after influenza A-associated pneumonia, while rivaroxaban was continued. Approximately 2 months later, the patient deteriorated and died from a presumed thromboembolic event. Although the precise cause of death could not be objectively confirmed, this post-discontinuation course is clinically noteworthy. Importantly, this temporal sequence should not be interpreted as evidence that ICI discontinuation directly caused recurrent thrombosis or death. The patient had advanced metastatic malignancy with a substantial baseline thrombotic burden and subsequently experienced severe infection, marked inflammatory activation, and acute kidney injury, all of which could independently have contributed to thrombo-inflammatory progression and clinical deterioration.

Several limitations should be acknowledged. This single-case observation cannot establish causality, and the limited number of longitudinal samples and absence of tissue factor, NET-associated, endothelial, and comprehensive functional immune profiling constrain mechanistic interpretation. In addition, influenza A-associated pneumonia, acute kidney injury, premature ICI discontinuation, and the unconfirmed cause of death represent important confounding factors. The subsequent renal deterioration during rivaroxaban therapy also complicates interpretation of the post-treatment thrombotic course.

Future prospective studies integrating tumor response, inflammatory markers, coagulation parameters, and immune profiling before, during, and after ICI treatment are warranted to determine whether thrombo-inflammatory changes reproducibly track immune-mediated tumor control and thrombotic risk in MSI-H hepatobiliary malignancies.

## 4 Conclusion

This case highlights the dynamic interplay between tumor-associated inflammation and cancer-associated thrombosis in MSI-H intrahepatic cholangiocarcinoma during PD-L1 blockade. The parallel changes in tumor burden, IL-6, and D-dimer suggest that immune checkpoint inhibition may influence thrombo-inflammatory activity in addition to tumor control. Although causality cannot be established from a single case, these findings provide a hypothesis-generating rationale for further investigation of the tumor–immune–coagulation axis in MSI-H hepatobiliary malignancies.

## Data Availability

The raw data supporting the conclusions of this article will be made available by the authors, without undue reservation.
